# The dual PI3K/mTOR inhibitor NVP-BEZ235 enhances nab-paclitaxel antitumor response in experimental gastric cancer

**DOI:** 10.3892/ijo.2013.2099

**Published:** 2013-09-13

**Authors:** CHANG-HUA ZHANG, NIRANJAN AWASTHI, MARGARET A. SCHWARZ, RODERICH E. SCHWARZ

**Affiliations:** 1 Division of Surgical Oncology, Department of Surgery, The University of Texas Southwestern Medical Center, Dallas, TX, USA; 2 Hamon Center for Therapeutic Oncology Research, Simmons Comprehensive Cancer Center, Dallas, TX, USA;; 3 Department of Pediatrics, The University of Texas Southwestern Medical Center, Dallas, TX, USA;; 4 Department of Gastrointestinopancreatic Surgery, The First Affiliated Hospital of Sun Yat-sen University, Guangzhou, Guangdong, P.R. China;; 5 IU Health Goshen Center for Cancer Care, Indiana University, Goshen, IN 46526, USA

**Keywords:** gastric cancer, PI3K/mTOR, BEZ235, nab-paclitaxel, chemotherapy

## Abstract

Gastric cancer is the second most common cause of cancer-related deaths worldwide. Taxanes have shown therapeutic effects against gastric cancer while also activating the PI3K/mTOR signaling pathway. We investigated the effects of NVP-BEZ235 (BEZ235), a novel dual PI3K/mTOR inhibitor, alone and in combination with nanoparticle albumin-bound (nab)-paclitaxel in experimental gastric cancer. Cell proliferation and protein expression were measured by WST-1 assay and immunoblotting. Tumor growth and survival studies were performed in murine xenografts. Phosphorylated mTOR and 4E-BP1 levels were elevated in gastric cancer cells and tumor tissues by nab-paclitaxel. BEZ235 effectively inhibited cell proliferation *in vitro* and provided additive effects in combination with nab-paclitaxel. Furthermore, BEZ235 blocked the activated PI3K/mTOR pathway either alone or in combination with nab-paclitaxel in gastric cancer cells. BEZ235 and nab-paclitaxel caused an increase in PARP-1 and caspase-3 cleavage. Net local tumor growth inhibition for the BEZ235, nab-paclitaxel and BEZ235+nab-paclitaxel groups was 45.1, 77.9 and 97% compared to controls. The effects of therapy on intratumoral proliferation and apoptosis corresponded with tumor growth inhibition data. BEZ235 also caused a decrease in phospho-mTOR and phospho-Akt in tumor tissue lysates. Median animal survival (controls, 23 days) was 26.5 days after BEZ235 (p=0.227), 90.5 days after nab-paclitaxel (p=0.001) and 97 days in the BEZ235+nab-paclitaxel combination treatment group (p=0.001). Our findings suggest that BEZ235 exerts some antitumor effects against gastric cancer and enhances effects of nab-paclitaxel through inhibition of cell proliferation and modulation of the PI3K/mTOR pathway. This approach may represent a promising combination targeted therapy for gastric cancer.

## Introduction

Gastric cancer (GC) is the fourth most prevalent cancer and the second most common cause of cancer-related deaths throughout the world (1–3). The prognosis of gastric cancer patients is generally poor with an overall 5-year survival of only ∼30–40% after radical resection. Current combination therapies with oxaliplatin and 5-fluorouracil carry limited efficacy but have the potential for considerable side effects (4–7). Development of chemoresistance is a common clinical phenomenon even in patients who have an initial positive clinical response to chemotherapy (8). Nanoparticle albumin-bound (nab) paclitaxel is a novel albumin-stabilized, cremophor-free and water-soluble nanoparticle formulation of paclitaxel (9). Clinical and experimental studies demonstrated that compared with solvent-based paclitaxel, nab-paclitaxel had higher tumor retention, lower toxicity (10,11) and more potent antitumor effects on ovarian cancer, melanoma, non-small cell lung carcinoma (NSCLC), breast cancer and pancreatic cancer (12–16a). We recently demonstrated that nab-paclitaxel showed stronger antitumor effects in experimental gastric cancer than contemporary commonly used cytotoxic agents such as oxaliplatin, epirubicin and docetaxel (16b). However, the greatest clinical tumor response rate to nab-paclitaxel is typically only 30–35% as seen in breast cancer (10) and incomplete responsiveness to nab-paclitaxel has also been observed in some tumors in our study. Thus, delineating the mechanism that restricts response and drives tumor resistance to nabpaclitaxel is important to improve its antitumor efficacy and explore more effective combination therapies.

The phosphatidylinositol-3-kinase (PI3K) and mammalian target of rapamycin (mTOR) signaling pathways play a central role for many tumor types in tumor cell proliferation, motility, invasion, metabolism and survival (17). A deregulated PI3K pathway is frequently encountered in gastric cancer (18,19) and it appears to play an important role in the aggressive nature of this disease while perhaps contributing to the lack of susceptibility to cytotoxic chemotherapy. Approximately 10% of gastric cancer patients carry a PIK3CA mutation, 20% a KRAS mutation and <2.7% a BRAF mutation (20,21). These mutations can activate PI3K and can lead to the activation of Akt via phosphorylation at Thr308 through PDK1 or/and at Ser473 through the mTOR associated with Rictor (the mTORC2 complex) (9). Activated Akt regulates key downstream effectors including mTOR associated with Raptor (mTORC1 complex), p70 S6 kinase and 4E-BP1 (10). Rapamycin and its analogous inhibit mTORC1 signaling and activate Akt signaling via mTORC2 related negative feedback loop. Thus, the combined inhibition of both PI3K and mTOR might be necessary for effective treatment of cancer.

NVP-BEZ235 (BEZ235), a novel dual PI3K/mTOR inhibitor and a synthetic small molecule of the class of imidazoquinolones, inhibits the catalytic subunit p110a of PI3K by competing at its ATP binding site and other class 1 PI3K enzymes and also inhibits the catalytic activity of mTOR (22,23). It was recently reported to mediate some anti-tumor effects in experimental pancreatic cancer (17), breast cancer (24) and gastric cancer (25). Phase I and II clinical trials of BEZ235 are currently under investigation in different solid tumors.

Nab-paclitaxel is a microtubule-stabilizing cytotoxic agent which causes mitotic arrest leading to cell death. Mechanisms of tumor resistance to taxanes are complicated and not completely elucidated. The Akt and mTORC1 pathways have shown to be activated after paclitaxel treatment (26–28). Inhibition of PI3Ks has been shown to sensitize tumors to paclitaxel, implying that PI3K inhibitors can regulate cell death in the presence of mitotic arrest (28–30). PI3K inhibitors could lead to an increase in lagging chromosomes, prolonged cell cycle arrest and cell death in prometaphase, while promoting nocodazole-induced mitotic cell death and reducing mitotic slippage (31). These results implied a mechanism of taxane-resistance associated with activation of the PI3K/mTOR pathway and provided a rationale for the evaluation of PI3K/mTOR inhibitors in combination with anti-mitotic drugs in order to improve cancer treatment outcomes.

In this study, we identified whether BEZ235 can down-regulate activation of Akt and mTOR in gastric cancer *in vivo* or *in vitro*. We also evaluated antitumor efficacy of BEZ235 alone and in combination with nab-paclitaxel in an attempt to determine a more effective gastric cancer therapeutic strategy.

## Materials and methods

### Cell culture and reagents

Human gastric cancer cell lines SNU16, NCI-N87 and AGS were obtained from the American Type Culture Collection (ATCC, Rockville, MD, USA). Cells were cultured in RPMI-1640 medium (Sigma Chemical Co. St. Louis, MO, USA) supplemented with 10% fetal bovine serum (FBS) in a humidified 5% CO_2_ atmosphere at 37°C. BEZ235 was purchased from LC Laboratories (Woburn, MA, USA) and nab-paclitaxel was purchased from Abraxis BioScience (Los Angeles, CA, USA). BEZ235 was dissolved in 1:9 NMP and PEG300. The cell proliferation reagent WST-1 was purchased from Roche Diagnostic Corp. (Indianapolis, IN, USA).

### Cell viability assay

Cell viability was evaluated by the colori-metric WST-1 assay. The measurement is based on the ability of viable cells to cleave the sulfonated tetrazolium salt WST-1 (4-[3-(4-iodophenyl)-2-(4-nitrophenyl)-2H-5-tetrazolio]-1,3-benzene disulfonate) by mitochondrial dehydrogenases (32). Gastric cancer cells (5,000 cells per well) were plated in a 96-well plate in regular growth medium and were treated with BEZ235, nab-paclitaxel, either alone or in combination at the ratio of their IC_50_ values (a series of 2-fold dilutions from 8 to 0.0625 times of IC_50_) after 16-h incubation. After an incubation of 72 h, 10 *μ*l of WST-1 reagent was added in each well followed by an additional incubation for 2 h. The absorbance at 450 nm was measured using a microplate reader.

### Median-effect analysis

Median-effect analyses were performed for combination assays of BEZ235 and nabpaclitaxel treatment according to the method of Chou and Talalay (33). Combination index (CI) values were plotted at each fraction affected (Fa) using CalcuSyn software (Biosoft) developed by Chou and Talalay. The CI is measured as a function of cells affected by the combined cytotoxic effect. A CI>1.1 indicates antagonism, while CI=0.9–1.1 indicates additivity and CI<0.9 indicates synergism.

### Immunocytochemical analysis

Gastric cancer cells (1×10^5^ cells per chamber) were plated in a 4-chamber slide in regular growth medium. After 24-h culture, cells were treated with nab-paclitaxel for 16 h and then fixed in 4% paraformaldehyde. Cells were then incubated with CAS blocking buffer followed by 1-h incubation with phospho-mTOR and phospho-4E-BP1 antibody (1:100) and 40-min incubation with Cy3 (1:200 dilution) secondary antibody. Slides were mounted using mounting solution containing 4′,6-diamidino-2-phenylindole (DAPI) (Invitrogen, Carlsbad, CA, USA). Fluorescence microscopy was used to detect fluorescent signals using the IX81 Olympus microscope equipped with a Hamamatsu Orca digital camera (Hamamatsu Corp., Bridgewater, NJ, USA).

### Western blot analysis

Subconfluent monolayers of cells were treated with BEZ235, nab-paclitaxel, either alone or in combination. Cell lysates and tumor lysates were obtained as previously described (34). Supernatants were recovered by centrifugation at 13,000 rpm, protein concentrations were measured and equal amounts of total protein were separated by SDS-PAGE. Proteins were transferred to PVDF membranes (Bio-Rad, Hercules, CA, USA) and the membranes were blocked for 1 h in TBS-T. The membranes were incubated overnight at 4°C with the following antibodies: p-Akt (Ser473), total Akt, p-mTOR (Ser2448), total mTOR, p-p70 S6K (Thr389), total p70 S6K, p-4E-BP1 (Thr37/46), total 4E-BP1, cleaved PARP-1, cleaved caspase-3 (all from Cell Signaling Technology, Beverly, MA, USA) and β-actin (Sigma, St. Louis, MO, USA). The membranes were then incubated with the corresponding HRP-conjugated secondary antibodies (Pierce Biotechnologies, Santa Cruz, CA, USA) for 1 h. Specific bands were detected using the enhanced chemiluminescence reagent (ECL, Perkin-Elmer Life Sciences, Boston, MA, USA) on autoradiographic film.

### Subcutaneous tumor growth study

All animal experiments were carried out in accordance with the guidelines and approved protocols of the University of Texas Southwestern Medical Center (Dallas, TX, USA) Institutional Animal Care and Use Committee (permit no. 2012-0081). Each animal was monitored daily throughout the experiment for any sign of distress. Female NOD SCID mice (6–8 weeks) were used for comparative modeling of subcutaneous tumor growth. Gastric cancer cells (20×10^6^ SNU16 cells) were subcutaneously injected into flank of each mouse. Mice were weighed twice a week. Fourteen days after tumor cell injection, all mice had measurable tumor with an average tumor size of 100–150 mm^3^. At this time-point, the animals were randomly grouped (n=6–8 per group) and treated intraperitoneally with PBS (control), BEZ235 (10 mg/kg, 3 times a week), nab-paclitaxel (10 mg/kg in 100 *μ*l PBS, 2 times a week), or BEZ235 (10 mg/kg, 2 times a week) combined with nab-paclitaxel (10 mg/kg in 100 *μ*l PBS, 2 times a week) for 14 days. The tumor size was measured twice weekly via caliper and tumor volume (V) was calculated by using the formula: V = ½ [L × (W)^2^], L = length and W = width. Relative tumor volume (RTV) was determined according to the formula RTV = V_n_/V_0_ where V0 represents the tumor volume at day 0 and V_n_ represents the tumor volume as measured after an interval of n days, respectively. Net growth in tumor size for each mouse was calculated by subtracting tumor volume on the first treatment day from that on the last day. After completion of treatment, all mice were euthanized with CO_2_ and tumors were excised, weighed and processed for histological, immunohistochemical and western blot analyses.

### Immunohistochemical analysis

Tumor tissue specimens were fixed in 4% paraformaldehyde and embedded in paraffin. Paraffin-embedded tissue sections were cut (5 *μ*m), deparaffinized, rehydrated and antigen retrieved. The tissue sections were incubated with CAS blocking buffer followed by 1-h incubation with 1:200 dilution of primary Ki67 (Abcam, Cambridge, MA, USA) or p-mTOR or p-4E-BP1 antibody (1:200) and 40-min incubation with Cy3 (1:200 dilution) secondary antibody. Slides were mounted using mounting solution containing DAPI (Invitrogen). Intratumoral apoptotic activity was evaluated by staining tissue sections with ‘ApopTag Apoptosis Detection kit’ according to the manufacturer’s (Millipore) instructions. Fluorescence microscopy was used to detect fluorescent signals using the IX81 Olympus microscope equipped with a Hamamatsu Orca digital camera (Hamamatsu Corp.) and a DSU spinning confocal unit using Slidebook software (Intelligent Imaging Innovations, Philadelphia, PA, USA). Intratumoral proliferative index and apoptotic index were evaluated by calculating positive cells in five high-power fields (HPF) per sample in a blinded manner.

### Animal survival analysis

Animal survival studies were performed using 6- to 8-week-old female SCID mice (35). The mice were intraperitoneally injected with SNU16 (40×10^6^) cells and body weight was measured twice a week. Two weeks after tumor cell injection mice were randomly grouped (6 to 7 mice per group) and treated intraperitoneally with PBS (control), BEZ235 (10 mg/kg, 2 times a week), nab-paclitaxel (10 mg/kg in 100 *μ*l PBS, 2 times a week), or BEZ235 (10 mg/kg, 2 times a week) combined with nab-paclitaxel (10 mg/kg in 100 *μ*l PBS, 2 times a week) for 2 weeks. Animal suffering was minimized by euthanizing when turning moribund according to predefined criteria including rapid weight loss or gain (>15%), failure to eat or drink, lethargy, or inability to remain upright. Animal survival was evaluated from the first day of treatment until death.

### Statistical analysis

GraphPad Prism 5 Software (GraphPad Software, San Diego, CA, USA) was used for analysis. Statistical analyses were performed by ANOVA for multiple group comparison and Student’s t-test for the individual group comparison. Survival group comparison was performed via log-rank test within a Kaplan-Meier type analysis. Values of p<0.05 were considered to represent statistically significant differences.

## Results

### Nab-paclitaxel increases phosphorylation of mTOR and 4E-BP1 in cultured gastric cancer cells and in gastric tumors in vivo

**Figure 1.**
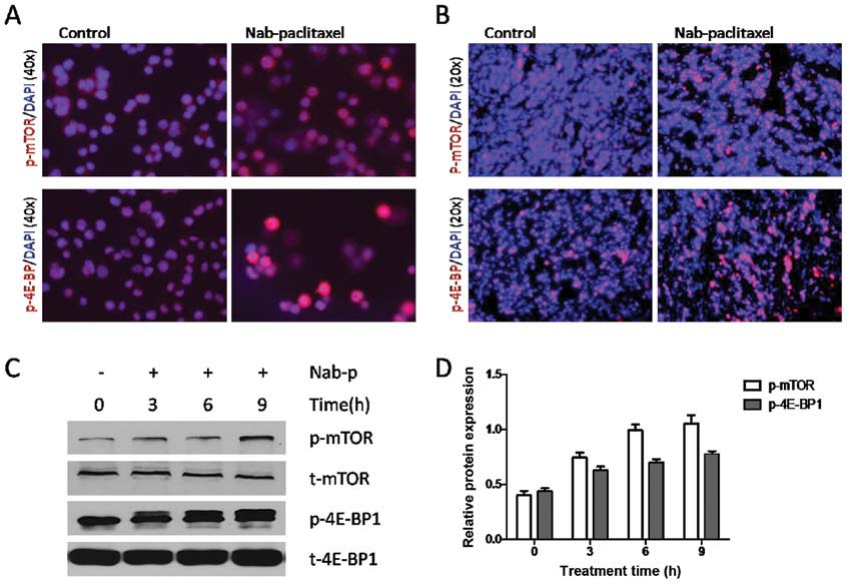
Nab-paclitaxel increases phosphorylation of mTOR and 4E-BP1 in cultured gastric cancer cells *in vitro* and in gastric tumors *in vivo*. (A) SNU16 cells were treated with nab-paclitaxel (10 *μ*M) for 16 h and showed increased expression of p-mTOR and p-4E-BP1 by immunocytochemical staining. (B) Tumors from SNU16 xenografted mice treated with nab-paclitaxel for 2 weeks were collected on the last day of the experiment and analyzed for the expression of p-mTOR and p-4E-BP1 by immunostaining tissue sections. (C) A subconfluent monolayer of SNU16 cells was treated with nab-paclitaxel (10 *μ*M) for 3, 6 and 9 h and analyzed by immunoblotting for p-mTOR, total mTOR, p-4E-BP1 and total 4E-BP1. (D) The intensity of bands was quantitated by densitometry and is represented in the bar graph as fraction of the total protein expression, respectively. Data are representative of three independent experiments with similar results.

Nab-paclitaxel treatment increased expression of p-mTOR and p-4E-BP1 in cultured SNU16 cells (Fig. 1A) and in SNU16 tumor tissues as observed by immunostaining (Fig. 1B). Western blot analysis results showed that nab-paclitaxel caused phosphorylation of mTOR and 4E-BP1 in a time-dependent manner (Fig. 1C and D). Similar results were observed in NCI-N87 cells and xenograft tumor tissues (data not shown).

### BEZ235 and nab-paclitaxel act additively in inhibiting gastric cancer cell proliferation

**Figure 2.**
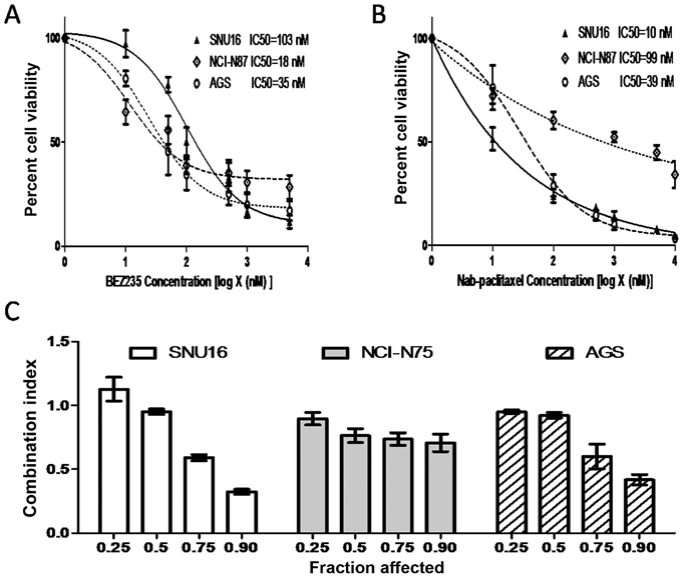
Additive effects of BEZ235 and nab-paclitaxel on cell proliferation *in vitro*. Human gastric cancer cells SNU16, NCI-N87 and AGS were plated on 96-well plates and treated with BEZ235 (A), nab-paclitaxel (B) and a combination of the two at a fixed ratio (C). After 72 h, 10 *μ*l WST-1 reagent was added in each well and incubated for 2 additional hours. The absorbance at 450 nm was measured using a microplate reader. The resulting number of viable cells was calculated by measuring absorbance of color produced in each well. The antiproliferative effect was then assessed and median effect analysis (CalcuSyn software) was used to evaluate the combination effect. The combination index (CI) is plotted as a function of the fraction of cells affected by the cytotoxic effect. CI>1.1 indicates antagonism, CI=0.9–1.1 indicates additivity and CI<0.9 indicates synergism. Data are the mean ± SD of triplicate determinations.

The cell proliferation inhibitory activity of BEZ235 and nab-paclitaxel in gastric cancer cells was measured using the WST-1 assay. BEZ235 and nab-paclitaxel inhibited cell proliferation in a dose-dependent manner. The IC_50_ of BEZ235 and nab-paclitaxel was 103 and 10 nM in SNU16, 18 and 99 nM in NCI-N87 cells and 35 and 39 nM in AGS, respectively (Fig. 2A and B). Median-effect analysis of BEZ235 in combination with nab-paclitaxel in SNU16, NCI-N87 and AGS cells is shown in Fig. 2C. Combination index (CI) values were <1.1 in SNU16 (except at the affected fraction level of 0.25), NCI-N87 and AGS cells, indicating the combinational effects of BEZ235 and nab-paclitaxel are synergistic to antagonistic in SNU16 cells and synergistic to additive in NCI-N75 and AGS cells (Fig. 2C).

### BEZ235 blocks PI3K/mTOR signaling proteins and induces apoptosis

**Figure 3.**
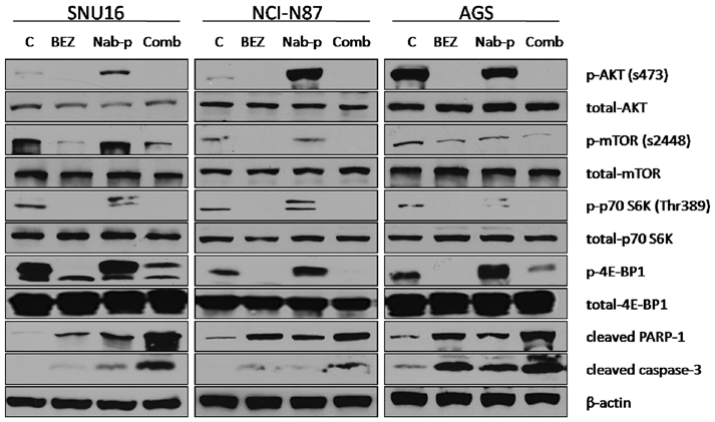
BEZ235 and nab-paclitaxel effects on the PI3K-mTOR signaling pathway and apoptosis-related proteins. Subconfluent monolayers of human gastric cancer cells SNU16, NCI-N87 and AGS were treated with PBS control (C), BEZ235 (10 *μ*M), nab-paclitaxel (10 *μ*M), or a combination for 16 h. Total cell extracts were analyzed by immunoblotting for p-Akt (Ser473), total Akt, p-mTOR (Ser2448), total mTOR, p-p70 S6K (Thr389), total p70 S6K, p-4E-BP1 (Thr37/46) and total 4E-BP1, cleaved PARP-1, cleaved caspase-3 and β-actin (loading control). Data are representative of two independent experiments with similar results.

The effect of BEZ235 on the PI3K/mTOR signaling pathway was investigated using SNU16, NCI-N87 and AGS gastric cancer cell lines. Immunoblot analysis revealed that BEZ235 blocked the expression of p-Akt, p-mTOR and phosphorylation of the downstream signaling proteins p70 S6K and 4E-BP1 in all three cells lines, while nab-paclitaxel increased phosphorylation of all these proteins in SNU16 and NCI-N87 cells. For AGS cells, nab-paclitaxel treatment increased expression of p70 S6K and 4E-BP1 but not of p-Akt and p-mTOR. BEZ235 in combination with nab-paclitaxel also blocked the expression of p-Akt and p-mTOR and phosphorylation of p70 S6K and 4E-BP1 in all three cells lines. The effect of BEZ235 on chemotherapy-induced apoptosis was also evaluated by analyzing cleavage of caspase-3 and PARP-1 proteins as markers of apoptosis. BEZ235 and nab-paclitaxel as single agent induced expression of cleaved caspase-3 and PARP-1, while the combination of BEZ235 with nab-paclitaxel led to additive effects on induction in cleavage of these apoptosis related proteins (Fig. 3).

### BEZ235 inhibits growth of SNU16 xenografts and enhances nab-paclitaxel antitumor response

**Figure 4.**
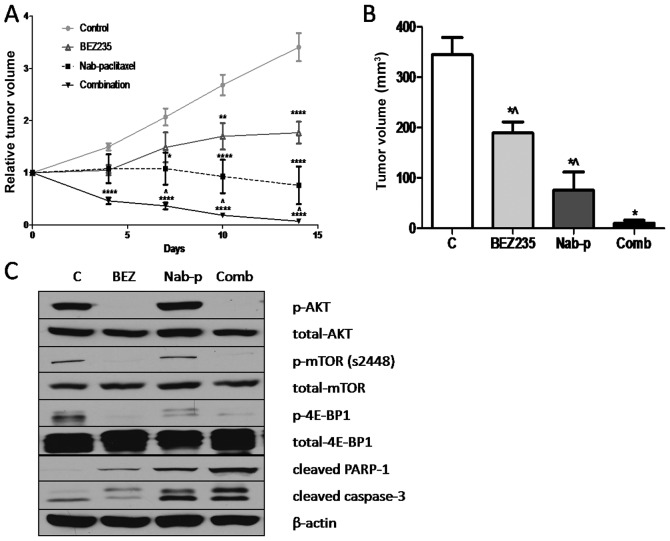
BEZ235 and nab-paclitaxel inhibit growth of established localized gastric tumor. SCID mice were subcutaneously injected with SNU16 cells (20×10^6^) and treated with BEZ235 and nab-paclitaxel for 2 weeks. (A) Relative tumor volume is calculated by dividing the tumor volume at any time by the tumor volume at the start of treatment. (B) Tumor volume was measured on the last day. Data are representative of mean values ± standard deviation from 6-8 mice per group. ^*, **, ****^Significant difference with p<0.05, p<0.01 and p<0.0001 versus control, respectively; ^^^significant differences (p<0.05) compared with combination therapy group. (C) BEZ235 blocks PI3K/mTOR signaling proteins and induces apoptosis-related proteins. Tumor lysates were prepared from tumor tissue samples obtained from SNU16 tumor bearing mice and were analyzed by immunoblotting. Data are representative of two independent experiments with similar results.

*In vivo* antitumor effects of BEZ235 were evaluated in a murine xenograft model using SNU16 cells. BEZ235 significantly inhibited the growth of SNU16 xenografts over the treatment time course of 14 days. Treatment of SNU16 tumor-bearing mice with BEZ235 resulted in statistically significant net tumor growth inhibition of 45.1% (p=0.0089), compared with the PBS treated control group (Fig. 4A and B). The evaluation of nab-paclitaxel alone treatment in this model resulted in net tumor growth inhibition of 77.9% (p=0.0011), compared with control. The combination treatment of SNU16 tumor-bearing mice with BEZ235 and nab-paclitaxel resulted in a 97% inhibition in net tumor growth (p<0.0001), compared with control group (Fig. 4A and B). Statistical analysis revealed that the difference in net tumor growth inhibition in the combination group was statistically significant compared with the nab-paclitaxel monotherapy (p= 0.034) or BEZ235 monotherapy (p<0.0001). No significant change in mouse body weight was observed after BEZ235, nab-paclitaxel or combination therapy.

Mechanisms of antitumor activity of BEZ235, either alone or in combination with nab-paclitaxel, were further examined by western blot analysis of protein lysates from SNU16 xenografts. BEZ235 treatment caused a significant decrease in expression of p-mTOR, p-Akt and p-4E-BP1. Evaluation of intratumoral apoptosis by analyzing expression of cleaved caspase-3 and cleaved PARP-1 proteins revealed that BEZ235 and nab-paclitaxel both induced cleavage of caspase-3 and PARP-1 and that combining these two agents had additive effects on cleavage of these apoptosis related proteins (Fig. 4C).

### BEZ235 inhibits intratumoral proliferation, induces apoptosis and enhances nab-paclitaxel response

**Figure 5.**
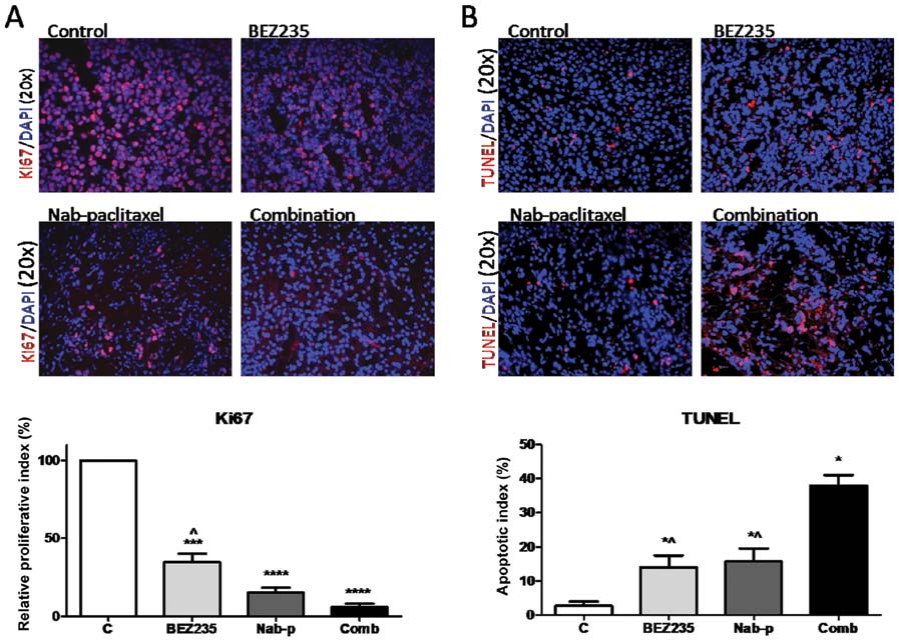
Effects of BEZ235 and nab-paclitaxel treatment on intratumoral proliferative and apoptotic activity. SCID mice were subcutaneously injected with SNU16 cells (20×10^6^) and treated with BEZ235 and nab-paclitaxel for 2 weeks. (A) Intratumoral proliferation was measured by immunostaining tissue sections with Ki67 nuclear antigen followed by fluorescence microscopy. Ki67-positive cells were counted in five high power fields per sample. Fold change in proliferative index was normalized compared to controls, with other samples being compared relative to this sample. (B) Intratumoral apoptosis was measured by staining tumor tissue section with the TUNEL procedure and subsequent fluorescence microscopy. The percentage of TUNEL-positive apoptotic cells was counted among five high power fields. For both immunostaining experiments, each group had at least three samples counted and the data are expressed as the mean ± standard deviation. ^*, ***, ****^Significant difference compared to control group with p<0.05, p<0.001 and p<0.0001, respectively; ^^^significant differences (p<0.05) compared with the combination therapy group.

Investigation of mechanisms of the antitumor activity of BEZ235 by immunohistochemical analyses of tumor tissues revealed that the tumors of BEZ235 treated mice presented a decreased tumor cell proliferation rate (Fig. 5A). Intratumoral proliferative index decreased by 65.1% (p=0.0003) in the BEZ235 treated group as compared to the control group. Nab-paclitaxel mono-therapy caused a 84.8% decrease in intratumoral proliferative activity compared with controls (p<0.0001). The combination of BEZ235 and nab-paclitaxel resulted in a 95% decrease in intratumoral proliferation compared with the control group (p<0.0001). The decrease in the intratumoral proliferative index in the combination treatment group was significantly higher than that after BEZ235 monotherapy (p=0.008), but not than that after nab-paclitaxel monotherapy (p=0.076).

Examination of intratumoral apoptosis in tumor tissues after BEZ235 and nab-paclitaxel treatment revealed that the increase in the apoptotic index was 5.1-fold in the BEZ235 monotherapy group (p=0.037) and 5.7-fold in the nabpaclitaxel treated group (p=0.032), compared with controls. The combination of BEZ235 and nab-paclitaxel had additive effects and a 13.2-fold enhanced intratumoral apoptosis was observed (p=0.004) compared to controls; this enhancement was statistically different from BEZ235 (p=0.006) or nabpaclitaxel (p=0.011) therapy alone (Fig. 5B).

### BEZ235 treatment enhances nab-paclitaxel survival benefits

**Figure 6.**
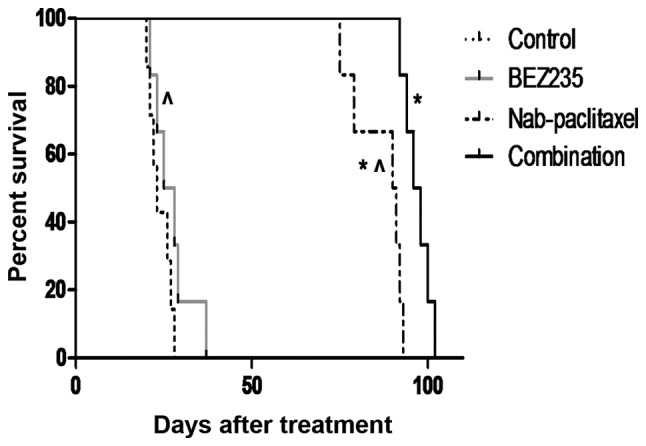
Effects of BEZ235 and nab-paclitaxel therapy on the overall survival of mice. SNU cells (40×10^6^) were injected intraperitoneally into SCID mice, followed after 2 weeks by treatment with BEZ235 (10 mg/kg, 2 times a week), nab-paclitaxel (10 mg/kg, 2 times a week) or a combination for a duration of 2 weeks. The curve represents the animal group survival time from the beginning of therapy. ^*^Significant difference compared to control group (p<0.05); ^^^significant differences (p<0.05) compared to the combination treatment group.

In an SNU16 murine peritoneal xenograft study, median survival of SCID-NOD mice was 23 days in the control group (Fig. 6). This median survival of mice was increased after BEZ235 treatment to 26.5 days (p=0.227 versus control group). Nab-paclitaxel monotherapy increased median survival to 90.5 days (p=0.0012 versus control group). Combination treatment with BEZ235 and nab-paclitaxel imposed additional effects on animal survival, as the median survival was prolonged to 97 days. Overall survival after combination therapy was significantly greater than that of the control group (p=0.0004) or the monotherapy groups (p=0.0022 versus nabpaclitaxel group and p=0.0005 versus BEZ235 group).

## Discussion

Gastric cancer represents a formidable treatment challenge as it frequently presents with metastatic disease upon diagnosis and a resulting high failure risk (36–38). Traditional double or triple cytotoxic chemotherapy regimens have limited therapeutic effects, but some considerable clinical side effects and a propensity towards the development of chemoresistance (39,40). We recently demonstrated that nab-paclitaxel has significantly stronger antitumor effects on gastric cancer *in vitro* and *in vivo* than other traditional cytotoxic compounds. The present study shows that nab-paclitaxel treatment increased phosphorylation of mTOR, Akt, p70 S6K and 4E-BP1 and that the novel dual PI3K/mTOR inhibitor BEZ235 was able to downregulate PI3K/mTOR signaling proteins either alone or in combination with nab-paclitaxel. BEZ235 inhibited cell proliferation *in vitro* and *in vivo* and still enhanced the already considerable antitumor response of nab-paclitaxel in gastric cancer xenografts. These data provide a rationale for combining BEZ235 and nab-paclitaxel therapies to sustain therapeutic benefits.

As discussed earlier, a deregulated PI3K pathway is frequently encountered in gastric cancer and can be considered one potentially targetable molecular driver of malignant progression. A recent study found that the dual PI3K/mTOR inhibitor BEZ235 reduced growth of NCI-N87 but not MKN45 and MKN28 gastric cancer xenografts, while tumor growth control did not correlate with PI3K/mTOR inhibition but thymidine kinase1 expression (25). BEZ235 was also found to be more effective against PIK3CA mutated AGS cells than the PIK3CA wild-type gastric cancer cells NCI-N87 and MKN-45 (41). For clinical solid tumor, the response rate was significantly higher for patients with PIK3CA mutations treated with PI3K/Akt/mTOR pathway inhibitors than for those without documented mutations (42). In the present study, BEZ235 turned out to be effective in inhibiting cell proliferation for all three human gastric cell lines, although SNU16 cells appeared to be less sensitive to BEZ235 than NCI-N87 or AGS cells. Irrespective of the *in vitro* responses, local tumor growth control and mouse survival studies demonstrated that BEZ235 monotherapy showed measurable effects in SNU16 xenograft models that correlated with intratumoral proliferative and apoptotic activity. Although, compared to nab-paclitaxel alone, the combination of BEZ235 with nab-paclitaxel is observed to increase mouse survival significantly, the median survival time is only slightly different than nab-paclitaxel alone, which might be due to the relatively high dose of nab-paclitaxel used or the relatively short duration of therapy. The cooperative interaction of nab-paclitaxel and BEZ235 may change by adjusting doses of each agent *in vivo*. From the results of BEZ235 obtained in SNU16 tumors we extrapolate that it should be effective also in NCI-N87 and AGS tumors. BEZ235, alone or in combination, blocked PI3K/mTOR pathway proteins in these three gastric cancer cell lines *in vitro* and in SNU16 xenograft tumor tissues. These findings support the expectation that the *in vivo* antitumor effects of BEZ235 are associated with inhibited functional activity of Akt, mTORC1 and mTORC2 and the related decrease in cell proliferation and induction of apoptosis in gastric cancer cell lines.

Classic anti-mitotic agents induce cancer cell death mainly through interfering with spindle assembly or disassembly and by thus increasing mitotic arrest. However, cancer cells can evade mitotic arrest before cell death, a mechanism that reduces the efficacy of conventional anti-mitotic drugs (43). A number of different groups have demonstrated that solvent-based paclitaxel activates Akt and mTORC1 signaling (26,27). Knocking down mTOR by shRNA decreased paclitaxel-induced Akt phosphorylation at Ser473 but not at Thr308 and it also caused CaOV3 ovarian cancer cells to become more sensitive to paclitaxel (30). Inhibition of PI3Ks promoted mitotic cell death, reduced mitotic slippage and improved the tumor killing effects of anti-mitotic drugs (31). PI3K is directly activated by loss of tumor suppressor PTEN or growth factor stimulation via the intracellular domain of a receptor tyrosine kinase. It may also be activated via stimulated GTPase RAS or by G-protein coupled receptors. In the present study, we found that nab-paclitaxel treatment increased phosphorylation of Akt, mTOR, p70 S6K and 4E-BP1, a mechanism that could enhance tumor cell-related resistance to nab-paclitaxel. Taxol treatment rapidly activates Akt signaling, while inhibition of taxol-induced PI3K/Akt signaling by the PI3K inhibitor Ly294002 decreases taxol-mediated survivin induction with a resulting enhancement of cell death (28). In our study, the dual PI3K/mTOR inhibitor BEZ235 blocked nab-paclitaxel-induced Akt and mTOR and managed to increase antitumor response of nab-paclitaxel. Additive effects of BEZ235 combined with nab-paclitaxel were demonstrated for both local tumor control and mouse survival extension. These observations can enhance the current level of understanding of certain molecular events of nab-paclitaxel response and escape, with identification of a rationale for a novel combination therapy for gastric cancer.

In conclusion, the present study on gastric cancer cells demonstrates that nab-paclitaxel activated components of the PI3K/mTOR pathway and that the dual PI3K/mTOR inhibitor BEZ235 alone or in combination with nab-paclitaxel was able to downregulate these PI3K/mTOR signaling proteins and to enhance apoptosis. BEZ235 and nab-paclitaxel combination had additive effects in local tumor control and resulted in survival improvement. These findings support the rationale for PI3K/mTOR targeted therapy in combination with nabpaclitaxel for gastric cancer.

## References

[1] Oh SC (2012). Update of adjuvant chemotherapy for resected gastric cancer. J Gastric Cancer.

[2] Schwarz RE, Smith DD (2007). Clinical impact of lymphadenectomy extent in resectable gastric cancer of advanced stage. Ann Surg Oncol.

[3] Jemal A, Bray F, Center MM, Ferlay J, Ward E, Forman D (2011). Global cancer statistics. CA Cancer J Clin.

[4] Pyrhonen S, Kuitunen T, Nyandoto P, Kouri M (1995). Randomised comparison of fluorouracil, epidoxorubicin and methotrexate (FEMTX) plus supportive care with supportive care alone in patients with non-resectable gastric cancer. Br J Cancer.

[5] Matsubara J, Shimada Y, Kato K (2011). Phase II study of bolus 5-fluorouracil and leucovorin combined with weekly paclitaxel as first-line therapy for advanced gastric cancer. Oncology.

[6] Boku N, Yamamoto S, Fukuda H (2009). Fluorouracil versus combination of irinotecan plus cisplatin versus S-1 in metastatic gastric cancer: a randomised phase 3 study. Lancet Oncol.

[7] Ohtsu A, Shah MA, Van Cutsem E (2011). Bevacizumab in combination with chemotherapy as first-line therapy in advanced gastric cancer: a randomized, double-blind, placebo-controlled phase III study. J Clin Oncol.

[8] Okada K, Fujiwara Y, Takahashi T (2013). Overexpression of forkhead box M1 transcription factor (FOXM1) is a potential prognostic marker and enhances chemoresistance for docetaxel in gastric cancer. Ann Surg Oncol.

[9] Elsadek B, Kratz F (2012). Impact of albumin on drug delivery - new applications on the horizon. J Control Release.

[10] Gradishar WJ, Tjulandin S, Davidson N (2005). Phase III trial of nanoparticle albumin-bound paclitaxel compared with polyethylated castor oil-based paclitaxel in women with breast cancer. J Clin Oncol.

[11] Gradishar WJ (2006). Albumin-bound paclitaxel: a next-generation taxane. Expert Opin Pharmacother.

[12] Coleman RL, Brady WE, McMeekin DS (2011). A phase II evaluation of nanoparticle, albumin-bound (nab) paclitaxel in the treatment of recurrent or persistent platinum-resistant ovarian, fallopian tube, or primary peritoneal cancer: a Gynecologic Oncology Group study. Gynecol Oncol.

[13] Kottschade LA, Suman VJ, Amatruda T (2011). A phase II trial of nab-paclitaxel (ABI-007) and carboplatin in patients with unresectable stage IV melanoma: a North Central Cancer Treatment Group Study, N057E (1). Cancer.

[14] Socinski MA, Bondarenko I, Karaseva NA (2012). Weekly nabpaclitaxel in combination with carboplatin versus solvent-based paclitaxel plus carboplatin as first-line therapy in patients with advanced non-small-cell lung cancer: final results of a phase III trial. J Clin Oncol.

[15] Volk LD, Flister MJ, Chihade D, Desai N, Trieu V, Ran S (2011). Synergy of nab-paclitaxel and bevacizumab in eradicating large orthotopic breast tumors and preexisting metastases. Neoplasia.

[16a] Von Hoff DD, Ramanathan RK, Borad MJ (2011). Gemcitabine plus nab-paclitaxel is an active regimen in patients with advanced pancreatic cancer: a phase I/II trial. J Clin Oncol.

[16b] Zhang C, Awasthi N, Schwarz MA, Hinz S, Schwarz RE (2013). Superior antitumor activity of nanoparticle albumin-bound paclitaxel in experimental gastric cancer. PLoS One.

[17] Awasthi N, Yen PL, Schwarz MA, Schwarz RE (2012). The efficacy of a novel, dual PI3K/mTOR inhibitor NVP-BEZ235 to enhance chemotherapy and antiangiogenic response in pancreatic cancer. J Cell Biochem.

[18] Byun DS, Cho K, Ryu BK (2003). Frequent monoallelic deletion of PTEN and its reciprocal associatioin with PIK3CA amplification in gastric carcinoma. Int J Cancer.

[19] Li VS, Wong CW, Chan TL (2005). Mutations of PIK3CA in gastric adenocarcinoma. BMC Cancer.

[20] Smyth EC, Cunningham D (2012). Targeted therapy for gastric cancer. Curr Treat Options Oncol.

[21] Wong H, Yau T (2012). Targeted therapy in the management of advanced gastric cancer: are we making progress in the era of personalized medicine?. Oncologist.

[22] Serra V, Markman B, Scaltriti M (2008). NVP-BEZ235, a dual PI3K/mTOR inhibitor, prevents PI3K signaling and inhibits the growth of cancer cells with activating PI3K mutations. Cancer Res.

[23] Maira SM, Stauffer F, Brueggen J (2008). Identification and characterization of NVP-BEZ235, a new orally available dual phosphatidylinositol 3-kinase/mammalian target of rapamycin inhibitor with potent in vivo antitumor activity. Mol Cancer Ther.

[24] Jegg AM, Ward TM, Iorns E (2012). PI3K independent activation of mTORC1 as a target in lapatinib-resistant ERBB2^+^ breast cancer cells. Breast Cancer Res Treat.

[25] Fuereder T, Wanek T, Pflegerl P (2011). Gastric cancer growth control by BEZ235 in vivo does not correlate with PI3K/mTOR target inhibition but with [^18^F]FLT uptake. Clin Cancer Res.

[26] Quintas-Cardama A, Verstovsek S (2013). Molecular pathways: Jak/STAT pathway: mutations, inhibitors and resistance. Clin Cancer Res.

[27] Van de Bor V, Zimniak G, Cerezo D, Schaub S, Noselli S (2011). Asymmetric localisation of cytokine mRNA is essential for JAK/STAT activation during cell invasiveness. Development.

[28] Xu R, Nakano K, Iwasaki H (2011). Dual blockade of phosphatidylinositol 3′-kinase and mitogen-activated protein kinase pathways overcomes paclitaxel-resistance in colorectal cancer. Cancer Lett.

[29] Kim SH, Juhnn YS, Song YS (2007). Akt involvement in paclitaxel chemoresistance of human ovarian cancer cells. Ann NY Acad Sci.

[30] Sun H, Yu T, Li J (2011). Co-administration of perifosine with paclitaxel synergistically induces apoptosis in ovarian cancer cells: more than just AKT inhibition. Cancer Lett.

[31] Hou H, Zhang Y, Huang Y (2012). Inhibitors of phosphatidylinositol 3′-kinases promote mitotic cell death in HeLa cells. PLoS One.

[32] Awasthi N, Schwarz MA, Verma V, Cappiello C, Schwarz RE (2009). Endothelial monocyte activating polypeptide II interferes with VEGF-induced proangiogenic signaling. Lab Invest.

[33] Chou TC, Talalay P (1984). Quantitative analysis of dose-effect relationships: the combined effects of multiple drugs or enzyme inhibitors. Adv Enzyme Regul.

[34] Awasthi N, Zhang C, Ruan W, Schwarz MA, Schwarz RE (2012). BMS-754807, a small-molecule inhibitor of insulin-like growth factor-1 receptor/insulin receptor, enhances gemcitabine response in pancreatic cancer. Mol Cancer Ther.

[35] Zhang C, Awasthi N, Schwarz MA, Schwarz RE (2013). Establishing a peritoneal dissemination xenograft mouse model for survival outcome assessment of experimental gastric cancer. J Surg Res.

[36] Zhang CH, Zhan WH, He YL, Chen CQ, Huang MJ, Cai SR (2007). Spleen preservation in radical surgery for gastric cardia cancer. Ann Surg Oncol.

[37] Xu J, Zhang C, He Y (2012). Lymphatic endothelial cell-secreted CXCL1 stimulates lymphangiogenesis and metastasis of gastric cancer. Int J Cancer.

[38] Schwarz RE, Zagala-Nevarez K (2002). Recurrence patterns after radical gastrectomy for gastric cancer: prognostic factors and implications for postoperative adjuvant therapy. Ann Surg Oncol.

[39] Hofheinz RD, Wenz F, Lukan N (2009). Oxaliplatin and capecitabine-based chemoradiotherapy for gastric cancer - an extended phase I MARGIT and AIO trial. Int J Radiat Oncol Biol Phys.

[40] Kobunai T, Watanabe T, Fukusato T (2011). Antitumour activity of S-1 in combination with cetuximab on human gastric cancer cell lines in vivo. Anticancer Res.

[41] Mueller A, Bachmann E, Linnig M (2012). Selective PI3K inhibition by BKM120 and BEZ235 alone or in combination with chemotherapy in wild-type and mutated human gastrointestinal cancer cell lines. Cancer Chemother Pharmacol.

[42] Janku F, Tsimberidou AM, Garrido-Laguna I (2011). PIK3CA mutations in patients with advanced cancers treated with PI3K/AKT/mTOR axis inhibitors. Mol Cancer Ther.

[43] Brito DA, Rieder CL (2006). Mitotic checkpoint slippage in humans occurs via cyclin B destruction in the presence of an active checkpoint. Curr Biol.

